# The Relationship between Self-objectification and Adolescent Appearance Anxiety: A Mediated Model with Moderation

**DOI:** 10.62641/aep.v53i2.1742

**Published:** 2025-03-05

**Authors:** He Zhang, Hongyu Li, Can Jiang, Yafeng Tu, Mengyao Xi

**Affiliations:** ^1^Department of Intercultural Studies, Kookmin University, 02707 Seoul, Republic of Korea; ^2^School of Preschool Education, Shangrao Preschool Education College, 334001 Shangrao, Jiangxi, China

**Keywords:** adolescent, self-objectification, social appearance comparison, self-compassion, appearance anxiety

## Abstract

**Background:**

The physical appearances of an individual are frequently scrutinized and evaluated by others in daily life. The rise of social media has intensified this scrutiny, leading to increased attention to and comparison of the appearance of an individual with others, leading to psychological challenges such as appearance anxiety. This study, from the perspective of self-objectification, explored the influence of self-objectification on appearance anxiety and the role of social appearance comparison and self-compassion in the relationship between self-objectification and appearance anxiety.

**Methods:**

A questionnaire survey was conducted among 842 adolescents using validated instruments, including the Self-objectification Scale, the Social Appearance Comparison Scale, the Self-compassion Scale, and the Appearance Anxiety Scale. Of these, 766 valid questionnaires were completed and analyzed.

**Results:**

(1) Self-objectification was a significant positive predictor of appearance anxiety. (2) Social appearance comparison partially mediated the relationship between self-objectification and appearance anxiety. (3) The second half of the mediation process in which self-objectification affects appearance anxiety through social appearance comparison was moderated by self-compassion.

**Conclusion:**

The effect of self-objectification on appearance anxiety is a mediated process with moderation.

##  Introduction

With the proliferation of the Internet, many individuals increasingly share aspects of their daily lives and physical appearances on social media. However, consumerism-driven marketing strategies have exaggerated narrow aesthetic ideals, such as the “A4 waist”, youthfulness, and thinness, through the promotion of beauty, makeup, fitness products and services. Media portrayal of celebrities and digitally enhanced images of internet personalities have contributed to rising appearance-related concerns, particularly among adolescent females, who may experience self-doubt and psychological challenges such as appearance anxiety [1]. Although the impact is less pronounced, adolescent males are also affected by the internalization of these media-driven ideals, leading to appearance anxiety among them [2]. Appearance anxiety has become a prevalent psychological challenge among adolescents, severely affecting their physical and mental health. This study aimed to explore the current state and underlying mechanisms of adolescent appearance anxiety and propose practical interventions to address this psychological concern.

### The Relationship between Self-objectification and Appearance Anxiety

Self-objectification, rooted in objectification theory [3], refers to the psychological process by which individuals internalize an external perspective of an observer, leading them to view and evaluate bodies as objects. Among females, experiences of sexual objectification contribute to self-objectification, which can lead to habitual monitoring of the body and appearance, and various negative psychological outcomes, such as increased shame and anxiety, ultimately affecting their mental health [4]. Appearance anxiety, a specific type of social anxiety, revolves around concerns about meeting societal aesthetic standards and the fear of negative evaluation based on physical appearance [5, 6]. Individuals with high levels of self-objectification are more likely to experience dissatisfaction with their appearance during self-monitoring, which leads to appearance anxiety [7]. Consequently, this study proposed hypothesis H1: Self-objectification is a significant positive predictor of appearance anxiety.

### The Mediating Role of Social Appearance Comparison in the Relationship between Self-objectification and Appearance Anxiety 

Festinger’s social comparison theory suggests that individuals have an inherent need to assess their abilities and perspectives, often by comparing themselves with others [8]. While the tendency for social comparison varies among individuals, those with a higher propensity for comparison engage more frequently [9]. Such frequent comparisons, especially against idealized standards, can lead to negative body perceptions and increased dissatisfaction [10]. This dissatisfaction, in turn, heightens anxiety when individuals feel that they do not meet societal standards [11]. Therefore, this study proposed hypothesis H2: Social appearance comparison mediates the relationship between self-objectification and appearance anxiety.

### The Moderating Role of Self-compassion in the Relationship between Self-objectification, Appearance Anxiety, and Social Appearance Comparison

Self-compassion is the ability to alleviate personal suffering by treating personal shortcomings, pain, and failures with openness and understanding, recognizing these experiences as part of the shared human conditions [12]. Individuals with high self-compassion are more likely to tolerate and accept their perceived inadequacies, even when social comparisons highlight them, thereby reducing negative emotions and promoting emotional resilience [13]. In contrast, those with low self-compassion tend to engage in negative self-evaluations, which can lead to increased anxiety and depression [14]. Consequently, individuals with higher levels of self-compassion may mitigate the negative effects of social appearance comparisons and appearance anxiety. Thus, this study proposed hypothesis H3: Self-compassion inversely moderates the relationship between social appearance comparisons and appearance anxiety.

Furthermore, as societal pressures on physical appearance have expanded beyond females to include males, and with the average age of sexual maturity decreasing to 12.5 years, self-objectification may also be occurring at younger ages. Previous research on objectification has primarily focused on female students, with limited attention to males and younger adolescents. This study, therefore, extends the investigation of self-objectification to include all adolescents.

**Fig. 1.**
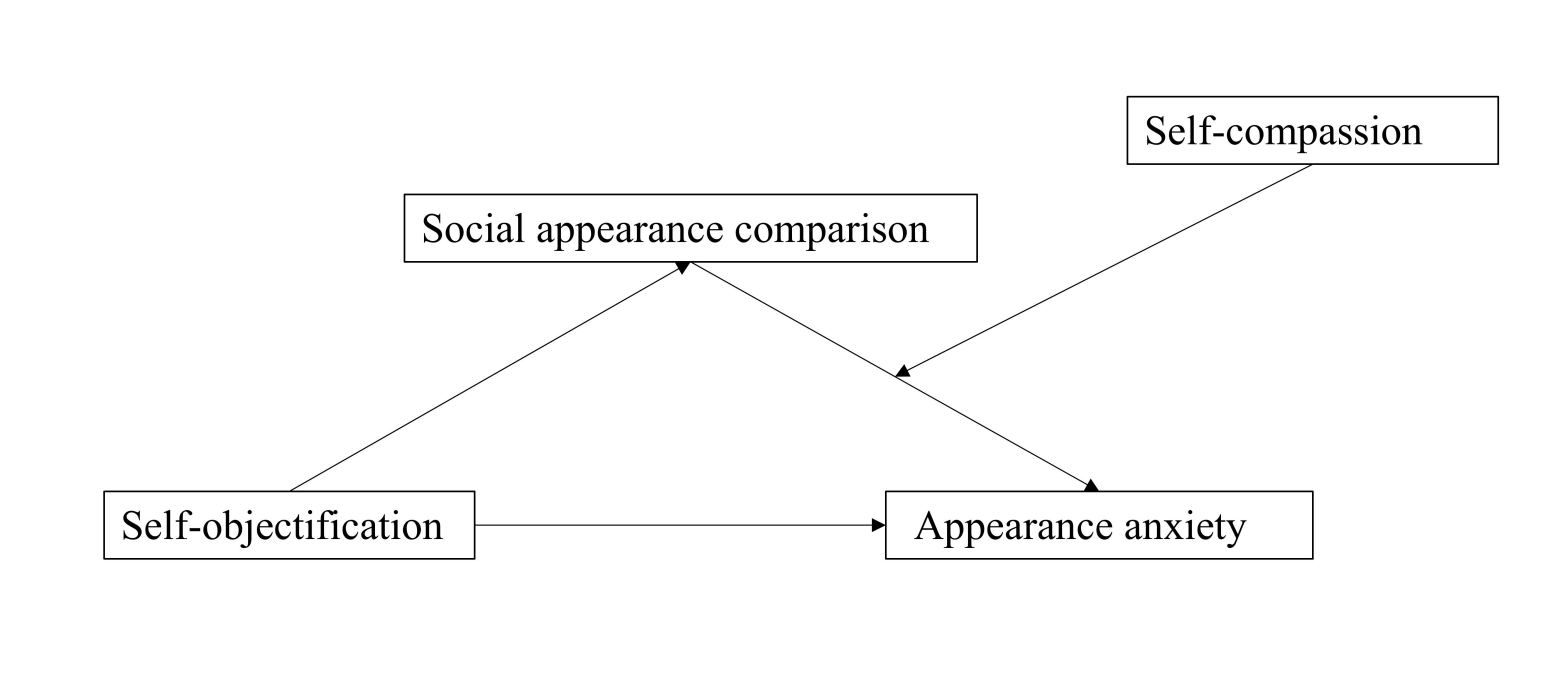
**Hypothetical model diagram of the relationship between self-objectification and appearance anxiety**.

In summary, this study explored the impact of self-objectification on appearance anxiety through a moderated mediation model. The model is shown in Fig. 1. The specific aims are: (1) To examine whether self-objectification positively predicts appearance anxiety. (2) To assess whether social appearance comparisons mediate the relationship between self-objectification and appearance anxiety. (3) To determine whether self-compassion moderates the latter part of the mediation process, where self-objectification affects appearance anxiety through social appearance comparisons.

## Research Methods

### Subjects

A total of 842 questionnaires were distributed to middle schools and universities in Jiangxi using whole-group sampling. Of these, 766 valid responses were obtained, yielding a validity rate of 90.97%. The sample included 322 males (42%) and 444 females (58%), with 243 junior high school students (31.7%), 280 high school students (36.6%), and 243 university students (31.7%). The mean age was 17.74 years (standard deviation = 4.60).

### Research Instruments

#### Self-objectification Scale

The Body Surveillance Subscale (BSS) from the Objectified Body Consciousness Scale (OBCS), developed by McKinley and Hyde [15], was used to assess the concerns of the participants about their appearance and body surveillance. The subscale consists of 8 items, with 2 positively scored and 6 negatively scored [15]. A 7-point scale was used, ranging from “1 = not at all” to “7 = fully”. Higher scores indicate greater concern for appearance. The internal consistency coefficient in this study was 0.705.

#### Social Appearance Comparison Scale

The Chinese version of the Physical Appearance Comparison Scale (PACS), revised by Chen Hong *et al*. [16], and five items from the Physical Comparison Scale (PCS), developed by Thompson [17], were used. The PCS was used to assess socially comparable traits related to physical appearance and consists of 5 items, with 4 positively scored and 1 negatively scored. A 5-point scale was used, with higher scores of “1 = never” and “5 = always” indicating a higher frequency of appearance comparisons. The internal consistency coefficient for this scale in the study was 0.853.

#### Self-compassion Scale

The Self-compassion Scale (SCS), developed by Neff [12] based on the 3 main components of self-compassion, and translated and revised by Chen Jian *et al*. [18], was used to measure self-compassion. The scale comprises 26 items and is scored on a 5-point scale, with higher total scores indicating a more objective view of the negative scenarios, a tolerant understanding and acceptance of individual shortcomings, and the recognition that negative experiences are common to all humans. The internal consistency coefficient of the scale in this study was 0.750.

#### Appearance Anxiety Scale

The Social Appearance Anxiety Scale (SAAS), developed by Hart *et al*. [19], was employed to measure appearance anxiety. The scale consists of 16 items with a 5-point scale ranging from “1 = not at all” to “5 = very much”. The total scores, derived from summing the item scores, reflect the overall appearance anxiety score of an individual, with higher scores indicating higher levels of appearance anxiety. The internal consistency coefficient of the scale in this study was 0.955.

### Data Analysis 

Data were analyzed using SPSS 25.0 (IBM, Armonk, NY, USA) and the PROCESS macro program by Hayes [20]. Model 14 in PROCESS was used for testing. Significance tests of regression coefficients were all performed using Bootstrap method (5000 repeated samples).

## Results

### Common Method Bias Test

Using Harman’s one-way test, all items of the questionnaire were subjected to an unrotated exploratory factor analysis, which resulted in eight factors with eigenvalues greater than one, with the first factor explaining 32.66% of the variance, which was less than the critical value of 40%. Therefore, no significant common method bias was detected in this study.

### Descriptive Statistics and Correlation Analysis 

Pearson product-moment correlations were conducted for self-objectification, social appearance comparison, self-compassion, appearance anxiety, and demographic variables. The results (Table 1) indicated that self-objectification was significantly positively correlated with social appearance comparison and appearance anxiety and significantly negatively correlated with self-compassion. Social appearance comparison was significantly negatively correlated with self-compassion and significantly positively correlated with appearance anxiety. Additionally, appearance anxiety was significantly negatively correlated with self-compassion.

**Table 1.** **Descriptive statistics and correlation analysis of variables (n = 766)**.

Variable	*M*	*SD*	1	2	3	4	5	6
Gender			1					
Grade			0.25^*⁣**^	1				
Self-objectification	3.93	0.98	0.04	0.16^*⁣**^	1			
Social appearance comparison	2.27	0.71	0.16^*⁣**^	0.16^*⁣**^	0.32^*⁣**^	1		
Self-compassion	3.17	0.40	–0.01	–0.01	–0.36^*⁣**^	–0.36^*⁣**^	1	
Appearance anxiety	2.23	0.89	0.03	–0.01	0.25^*⁣**^	0.62^*⁣**^	–0.45^*⁣**^	1

**Note**: ^*⁣**^p < 0.001. *SD*, standard deviation.

### Moderated Mediation Analysis of Self-objectification and Appearance Anxiety

According to the method of Wen Zhonglin and Ye Baojuan [21], all predictor variables were standardized before data analysis, and gender and age factors were also controlled. The results are summarized in Table 2. In Model 1, self-objectification significantly predicted social comparison. In Model 2, self-objectification and social appearance comparison significantly predicted appearance anxiety. In Model 3, the direct effect of self-objectification on appearance anxiety became insignificant with the addition of the moderator variable. Social appearance comparison and self-compassion remained significant predictors of appearance anxiety, and the interaction term between social appearance comparison and self-compassion was also a significant predictor. These findings indicate that self-compassion moderates the relationship between social appearance comparison and appearance anxiety, particularly in the second stage of the mediation process: self-objectification → social appearance comparison → appearance anxiety.

**Table 2.** **Mediation analysis of the moderating role of self-compassion in the relationship between self-objectification and appearance anxiety**.

	Model 1 (social appearance comparison)		Model 2 (appearance anxiety)		Model 3 (appearance anxiety)	
	β	*t*	β	*t*	β	*t*
Gender	0.25	3.56^*⁣**^	–0.09	–1.50	–0.08	–1.53
Grade	0.09	2.13^*^	–0.12	–3.80^*⁣**^	–0.10	–3.20^**^
Self-objectification	0.31	8.96^*⁣**^	0.07	2.43^*^	0.01	0.27
Social appearance comparison			0.62	20.61^*⁣**^	0.53	17.24^*⁣**^
Self-compassion					–0.26	–8.55^*⁣**^
Social appearance comparison × self-sympathy					–0.08	–3.34^*⁣**^
R^2^	0.13		0.40		0.46	
*F*	38.14^*⁣**^		127.93^*⁣**^		107.59^*⁣**^	

**Note**: ^*^p < 0.05, ^**^p < 0.01, ^*⁣**^p < 0.001.

**Fig. 2.**
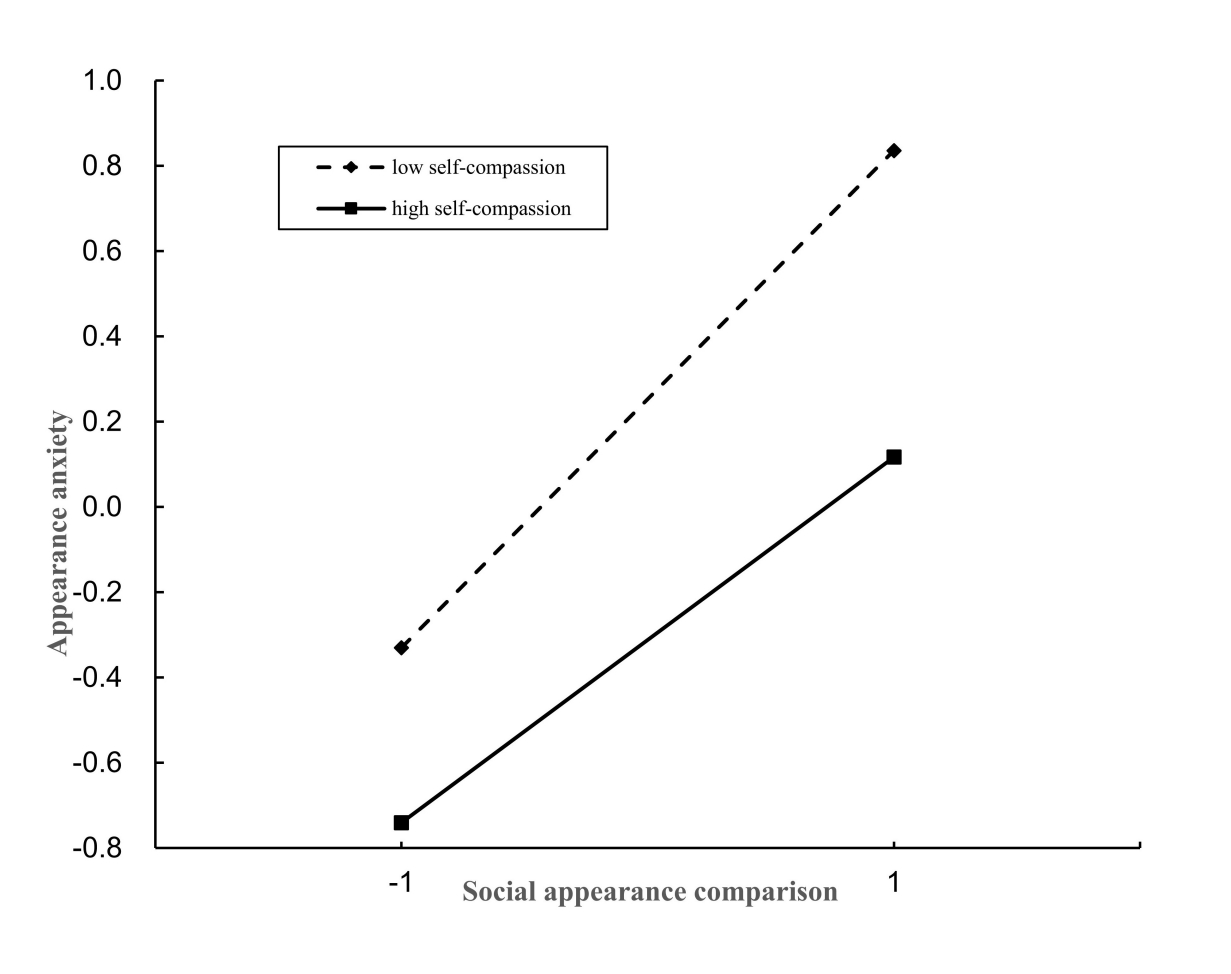
**Moderating effect of self-compassion on the relationship between social appearance comparison and appearance anxiety**.

Plus or minus one standard deviation from the mean was used to define high and low self-compassion groups. Simple slope analyses were then performed. As shown in Fig. 2, social appearance comparisons significantly predicted appearance anxiety in the low self-compassion group (b = 0.60, *t* = 17.95, *p* < 0.001). However, in the high self-compassion group, the predictive effect of social appearance comparisons on appearance anxiety was significantly weaker (b = 0.45, *t* = 10.80, *p* < 0.001).

## Discussion

This study confirmed that self-objectification is a positive predictor of appearance anxiety, indicating that higher levels of self-objectification increase the likelihood of experiencing appearance anxiety. This finding is consistent with previous studies [22, 23, 24]. Individuals with high self-objectification often view themselves from an external perspective, frequently monitor their physical appearance, and are more prone to anxiety when their appearance does not align with societal expectations.

Additionally, the study found that social appearance comparisons partially mediated the relationship between self-objectification and appearance anxiety. Self-objectification directly influences appearance anxiety and indirectly affects it through social appearance comparisons. These findings support the idea that self-objectification can lead to lower appearance satisfaction through social appearance comparison [25]. By exploring the mechanism of self-objectification on appearance anxiety, it was found that self-objectification, as a way of perceiving the bodily self, has similarities with body imagery, and it may affect the bodily self through internalization and social comparison [26]. Individuals with high self-objectification are more likely to evaluate their appearance by frequently comparing themselves to others, seeking a relative standard due to the absence of an absolute benchmark for physical appearance [27]. This constant comparison, particularly among those prone to social comparison tendency, often results in increased appearance anxiety when they perceive themselves as falling short [28, 29].

Moreover, this study explored the moderating role of self-compassion in the relationship between self-objectification, social appearance comparisons, and appearance anxiety. Self-compassion was found to moderate the latter part of the mediation pathway, specifically in the impact of social appearance comparisons on appearance anxiety. Self-compassion, defined as the ability to empathize with oneself in the face of personal shortcomings [12], was significantly negatively associated with appearance anxiety, body dissatisfaction, and body shame [30, 31]. Intervention studies have further demonstrated that self-compassion helps individuals accept their imperfections, reducing body shame and anxiety [32, 33].

Social comparisons can be categorized into two types based on modality: difference and similarity comparisons, and two types based on direction: upward and downward comparisons [34]. Self-objectifying women often engage in differential comparisons, which are more likely to result in negative body perceptions compared to similarity comparisons [35]. When these women perceive their appearance as inadequate, it can lead to negative self-perception and heightened appearance anxiety.

Combining modality and direction, it was found that individuals with low self-compassion tend to make upward differential comparisons, lowering their self-evaluation and increasing body dissatisfaction, thus leading to appearance anxiety. In contrast, individuals with high self-compassion, even when engaging in frequent differential comparisons, are more likely to make downward comparisons. They compare themselves with those who are at a lower level, viewing their appearance shortcomings as common and acceptable, which helps them forgive and understand themselves. Downward comparisons in this context enhance self-evaluation [36]. High self-compassionate individuals approach their physical appearance with openness and tolerance. When they identify deficiencies in their appearance, they understand that their appearance may not meet societal standards, but they find this acceptable, which helps them resolve negative emotions and reduce their anxiety. Thus, increasing self-compassion is crucial for adolescent mental health and can effectively alleviate appearance anxiety, even in the context of sexual objectification.

However, this study has limitations. First, due to the cross-sectional nature of the data, it is difficult to establish causal relationship between variables. Future studies could use longitudinal tracking or experimental methods to verify these relationships. Second, the sample size is limited, with participants primarily from a single province in China. Self-objectification and appearance anxiety may vary across different economic and cultural backgrounds. Future studies should explore adolescent appearance anxiety from broader cultural and social perspectives.

## Conclusion

Self-objectification is a significant predictor of appearance anxiety. Social appearance comparison mediates the relationship between self-objectification and appearance anxiety. The latter half of the mediating process, where self-objectification affects appearance anxiety through social appearance comparison, is moderated by self-compassion.

## Availability of Data and Materials

The data used to support the findings of this study are available from the corresponding author upon request.
